# Polymorphism profiling of nine high altitude relevant candidate gene loci in acclimatized sojourners and adapted natives

**DOI:** 10.1186/s12863-015-0268-y

**Published:** 2015-09-15

**Authors:** Arvind Tomar, Seema Malhotra, Soma Sarkar

**Affiliations:** Defence Research and Development Establishment, Ministry of Defence R&D Organization, Jhansi Road, Gwalior, 474002 India; Defence Institute of Physiology and Allied Sciences, Ministry of Defence R&D Organization, Lucknow Road, Delhi, 110054 India

**Keywords:** Polymorphism, Sea level sojourners, Acclimatized, Adapted, High altitude natives, Ladakh, Indian

## Abstract

**Background:**

Sea level sojourners, on ascent to high altitude, undergo acclimatization through integrated physiological processes for defending the body against oxygen deprivation while the high altitude natives (resident population) are adapted to the prevailing hypobaric hypoxic condition through natural selection. Separating the acclimatization processes from adaptive changes and identifying genetic markers in lowlanders that may be beneficial for offsetting the high altitude hypoxic stress, although challenging, is worth investigating. We genotyped nine candidate gene polymorphisms, suggested to be relevant in high altitude environment, in sea level acclimatized sojourners and adapted natives for understanding differences/commonality between the acclimatized and the adapted cohorts at the genetic level.

**Results:**

Statistically similar genotypic and allelic frequencies were observed between the sea level sojourners (acclimatized) and the high altitude natives (adapted) in six loci viz., *EDN1 (*endothelin 1) -*3A/-4A VNTR*, *ADRB2 (*beta-2 adrenergic receptor, surface) *Arg16Gly (rs1042713:A > G), ADRB3 (*beta-3 adrenergic receptor) *Trp64Arg (rs4994:T > C), eNOS* (nitric oxide synthase, endothelial) *Glu298Asp (rs1799983:T > G)*, *TH* (tyrosine hydroxylase) *Val81Met (rs6356:G > A)* and *VEGF* (vascular endothelial growth factor) *963C > T (rs3025039:C > T)* while *SCNN1B* (amiloride-sensitive sodium channel, subunit beta) *Thr594Met (rs1799979:C > T)* was monomorphic. Genotypic and allelic frequencies in *EDN1 9465G > A (rs2071942:G > A)* and *ADRB2 Gln27Glu (rs1042714:G >* C) were significantly different between the acclimatized sojourners and the high altitude natives with higher frequency of *GG* and *GA* genotypes of *EDN1 rs2071942* and *CC* genotype of *ADRB2 rs1042714* being observed in Ladakh natives. Mutated *A* allele (*AA* genotype) of *rs2071942* and carriers of *G* allele (*GG* + *GC* genotypes) of *rs1042714* were less favorable during acclimatization under recessive and dominant genetic models of inheritance respectively indicating thereby that *GG* genotype and *G* allele of *EDN1 rs2071942* and *CC* genotype of *ADRB2 rs1042714* conferred acclimatization benefit.

**Conclusion:**

Sea level acclimatized individuals shared similarity with the adapted natives in certain high altitude relevant genetically based trait variation suggesting advantageous consequence as well as commonality in gene regulatory pathways in which these gene products function both during process of acclimatization and adaptation in high altitude environment.

**Electronic supplementary material:**

The online version of this article (doi:10.1186/s12863-015-0268-y) contains supplementary material, which is available to authorized users.

## Background

High altitude regions (terrestrial elevation >3000 meters) are visited by sojourners, recreational travelers, mountaineers, climbers, trekkers, personnel on duty and many others irrespective of encountering hardships of whole body hypoxic stress and threat to health as high altitude illness. The sea level sojourners, on arrival to high altitude, undergo acclimatization (a progressive tolerance to hypoxia through a series of complex and finely integrated physiological process mostly comprising of cardiopulmonary and hematological responses) for mitigating the reduced oxygen partial pressure and defending the physiological responses against oxygen deprivation [1]. Nevertheless, even after initial acclimatization, there is reduction in physical work capacity with fall in arterial oxygen saturation, decrease in maximal heart rate and reduction in maximum oxygen uptake capacity (VO_2max_) [2–4]. Natives from the high altitude regions like the Tibetan plateau in the Himalayas (with long term resident populations of Tibetans, Ladakhis and Sherpas), the Andean *altiplano* (with resident populations of Quechua and Aymara), the Semien Plateau of North Africa (with resident population of Ethiopians), Tien-Shan and Pamir mountains in Asia (populated by the Kyrgyz) have developed distinctive patterns of adaptation to the high altitude environment [5, 6] with biological characteristics and genetic selection that off set high altitude hypoxic stress [7, 8]. While adaptation involves changes that take place over generations of natural selection enabling the body to function better at high altitude, acclimatization is a reversible physiological phenomenon aimed at protecting the body from hypoxic stressor.

The high altitude natives from Ladakh (which is the highest plateau in the trans Himalayan region of the Indian state of Jammu and Kashmir, terrestrial elevation ~ 3800–4000 m) are adapted to the high altitude hypoxic environment and have higher VO_2max_ [9], larger lung volume and capacity [10] and significantly higher redox status [11] compared to the acclimatized sojourners. It is obvious that high altitude natives of Ladakh would have features of hypoxic adaptation at the genetic level although such information is sparse. Limited comparative studies of genetic profiles between the high altitude natives of Ladakh and the sea level sojourners reported predominance of insertion (*I*) allele of angiotensin converting enzyme (*ACE)* gene in the Ladakh natives compared to sea level sojourners [12]. The insertion (*I*) allele has been suggested to be associated with lower ACE protein function while deletion (*D*) allele is responsible for elevated ACE activity [13, 14]. Distribution of *I* dominant genotype and *I* allele was reported to be significantly higher in the Sherpas [15]. In Peruvians, *ACE I/I* genotype was shown to associate with higher resting and submaximal exercise arterial oxygen saturation (SaO_2_) indicating central cardiopulmonary effect of *ACE I* allele with ventilation and SaO_2_ [16]. *ACE I/D* locus is either functionally related to arterial oxygen saturation (SaO_2_) or is in close linkage disequilibrium with a true causal locus affecting SaO_2_ at high altitude wherein inheritance of *I* allele along with the allelic variant at the causal locus would increase SaO_2_ while inheritance of *D* allele along with the allelic variant at the causal locus would decrease SaO_2_ [16]. Overrepresentation of *I* allele of *ACE* gene might be one of the fundamental genetic factors responsible for maintaining physiological low ACE activity at high altitude, thereby playing an advantageous physiological role in adapting to a high altitude environment and providing an edge for beneficial adaptation/acclimatization to high altitude. Interestingly the *D* allele of *ACE* gene was seen to be associated with high altitude pulmonary edema in Indian population in a recent study [17]. ACE converts angiotensin I, which is generated by enzymatic cleavage of angiotensinogen, to angiotensin II. Studies have suggested role of human renin-angiotensin-aldosterone systems (RAAS) in high altitude hypoxic adaptation [18]. Level of angiotensinogen, which is a glycoprotein and coded by Angiotensinogen gene (*AGT*), relates directly to blood pressure and is modified by AGT gene variants [19]. Significant difference in genotype and allele frequency of *Thr174Met* (*rs4762*) polymorphism in angiotensinogen (*AGT*) gene was observed between Ladakh natives and acclimatized sojourners while *A1166C* (*rs5186*) polymorphism in angiotensin II type I receptor (*AGTR1)* gene and *C-344 T* (*rs1799998*) polymorphism in aldosterone synthase (*CYP1B2)* gene was found to be similar between the two cohorts in an earlier study [20].

Circulating level of nitric oxide (NO), which is a vasodilator, has been shown to be higher in the high altitude Ladakh population compared to lowlanders [21]. Generation of high level of NO and circulating nitrogen oxide species have been suggested to enable greater blood flow and oxygen delivery to offset hypoxia [22] thereby being beneficial in high altitude environment. NO is catalyzed by nitric oxide synthase (*NOS*) gene and its role in adaptation at high altitude and etiology of high altitude disorder [23] is well documented. Higher production of NO was reported to be associated with over representation of *Glu* allele of *Glu298Asp* variant of nitric oxide synthase (endothelial) gene (e*NOS)* in the Ladakh natives [21]. Reduced plasma level of endothelin associated with over expression of endothelin 1 (*EDN1)* genotypes -*3A/-3A*, *GG* and *Lys198Lys* was also observed in high altitude Ladakh population compared to sea level sojourners [24]. Endothelin, which is a vasoconstrictor hormonal peptide encoded by endothelin 1 (*EDN1*), is stimulated under hypoxic environment [25] and suggested as a potential drug target for prophylactic measures at high altitude [26]. Endothelin-1 is considered to be a major factor in development of hypoxic pulmonary hypertension [27] which could ultimately lead to high altitude pulmonary edema [28] by augmenting capillary hydrostatic pressure in susceptible individual [29]. The proximal promoter of *EDN1* contains hypoxia inducible factor 1 (HIF1) binding site [30].

Separating the adaptive changes from acclimatization processes and identifying genetic markers in lowlanders that may be beneficial for offsetting the high altitude hypoxic stress, although challenging, is worth investigating. We hypothesized that there may be similarity in genetically based trait variation between the acclimatized and adapted individuals, protein products of which would be advantageous to mitigate environmentally induced physiological responses during exposure to high altitude hypoxia. We chose nine candidate gene polymorphisms from published literature based on their suggested relevance in high altitude environment and studied them in sea level sojourners who acclimatized to the high altitude compared to the high altitude adapted Ladakh natives. The variants selected for study were *EDN1* (endothelin 1) *9465G > A (rs2071942:G > A), EDN1 -3A/-4A VNTR, ADRB2* (beta-2 adrenergic receptor, surface) *Gln27Glu (rs1042714:G > C), ADRB2 Arg16Gly (rs1042713:A > G), ADRB3* (beta-3 adrenergic receptor) *Trp64Arg (rs4994:T > C), eNOS* (nitric oxide synthase, endothelial) *Glu298Asp (rs1799983:T > G)*, *SCNN1B* (sodium channel, non voltage gated 1 beta subunit) *Thr594Met (rs1799983:G > T), TH* (tyrosine hydroxylase) *Val81Met (rs6356:G > A)* and *VEGF* (vascular endothelial growth factor) *963C > T (rs3025039:C > T).* Beta-2 adrenergic receptor (encoded by *ADRB2)* is a dominant receptor subtype in lungs [31]. Role of ADRB2 in regulating lung fluid clearance is well defined [32] and improved O_2_ delivery during acclimatization at high altitude through adrenergic contribution is reported [33]. *ADRB2* gene polymorphism is also shown to affect vasodilation [34, 35]. Amiloride-sensitive sodium channel subunit beta, encoded by *SCNN1B* is expressed in aldosterone-responsive epithelial cells of kidney and colon and plays a critical role in control of sodium balance, blood volume and blood pressure [36]. It has a distinct role in lung in controlling the ionic composition of the air-liquid interface and rate of mucociliary transport. Down regulation of SCNN1 and Na^+^,K^+^-ATPase functions are observed to impair alveolar fluid clearance under hypoxia [37]. Tyrosine hydroxylase (*TH*) is a hypoxia-induced enzyme that sets the synthesis rate of the neurotransmitter dopamine in the carotid body (the chemo sensitive organ) and regulates the ventilator response to hypoxia [38]. *In vivo* up regulation of *TH* gene in the caudal brain stem is associated with adaptive ventilator mechanism essential for respiratory homeostasis during hypoxia [39, 40]. High altitude exposure has also been shown to have an effect on vascular endothelial growth factor level in man [41]. Vascular endothelial growth factor (*VEGF*) is an angiogenesis-related gene from the vascular endothelial growth factor signaling pathway. It is a potent permeability factor regulated by hypoxia and an important component for pathogenesis of high altitude adaptation and sickness [41, 42].

## Methods

### Study participants and sample collection

A total of 151 healthy male unrelated Army recruits of Indian origin comprising 69 high altitude natives from Ladakh (mean age 28.7 ± 6.2) and 72 acclimatized sea level sojourners (mean age 28.3 ± 6.7) participated in the study. The high altitude natives of Ladakh (adapted to the high altitude hypoxic environment) from Indian Army’s Ladakh Scout comprised mostly of local Tibetan commandoes. The sea level sojourners were from the Northern and Southern plains of India who were assigned to high altitude duties. The sojourners were air lifted from Delhi (sea level) to Leh, Ladakh (altitude 3500 m) and completed a Lake Louise acute mountain sickness self-assessment score [43] within 24–48 h after arrival at high altitude to rule out acute mountain sickness. The sojourners were rested for two days for acclimatization before blood collection. The volunteers were part of an ongoing high altitude research program of the Defence Institute of Physiology and Allied Sciences and gave written informed consent prior to their inclusion in the study. The study protocol was approved by the ‘Institute Ethics Committee of Defence Institute of Physiology and Allied Sciences’ (DIPAS), Delhi. 3–4 ml venous blood was collected from the volunteers in the morning in seated position. Subjects abstained from smoking for 12 h before sample collection. The volunteers were not on medication.

### Physiological measurements

Physiological variables of age, body weight, heart rate (HR), blood pressure and arterial oxygen saturation (SaO_2_) were measured. SaO_2_ and heart rate (HR) were measured in the natives and the acclimatized volunteers by Finger Pulse Oximeter (Nellcor N-20P, Nellcor Puritan Benett Ltd, UK) on warm hands in seated position. During recording, the volunteers breathed room air. SaO_2_ values were recorded after they remained constant for at least one minute.

### Genotyping

We genotyped nine polymorphisms from seven genes in 151 participants. Brief description of the studied polymorphisms is given in Table 1. Primers were checked for sequence specificity using Blast Local Alignment Search Tool (http://blast.ncbi.nlm.nih.gov/Blast.cgi). DNA samples had a A_260_/A_280_ ratio of 1.8-1.9, and were adjusted to 20 ng/μl. A total of approximately 150 ng of genomic DNA was used for PCR reaction. Table 2 summarizes the primer sequences, annealing temperatures and size of the restricted fragments. PCR reactions were run on Gene Amp PCR System 9700 (Applied Biosystems). Of the 1359 possible determinations, 99.19 % were successfully genotyped. Genotyping errors were ruled out by replicating the experiments. Genotypes were randomly validated on 3100 DNA Sequencer (Applied Biosystems).

**Table 1:** Brief description of the studied polymorphisms

Gene	Chromsomal region	Variation class	Variant	NCBI rsID	Function	Reference SNP allele	Ancestral allele	Residue change	Allele change	Minor allele	MAF^a^
*EDN1*	6p24.1	SNV	9465G > A	*rs2071942*	Intronic	A/G	A	na	na	A	0.255
*EDN1*	6p24.1	DIV	3A/4A (134delA)	*rs1800997*	UTR-5	-/A	A	na	na	A	0.216
*SCNN1B*	16p12.2	SNV	Thr594Met (1781C > T)	*rs1799979*	Missense	C/T	C	T[Thr] = > M[Met]	A*C*G = > A*T*G	T	0.003
*ADBR2*	5q31-q32	SNV	Arg16Gly (46A>G)	*rs1042713*	Missense	-G/A	G	R[Arg] = > G[Gly]	*A*GA = > *G*GA	A	0.47
*ADRB2*	5q31-q32	SNV	Gln27Glu (79G>C)	*rs1042714*	Missense	C/G	G	Q[Gln] = > E[Glu]	*C*AA = > *G*AA	G	0.238
*ADBR3*	8p12-p11.1	SNV	Try64Arg (5387 T > C)	*rs4994*	Missense	C/T (rev)	C	W[Trp] = > R[Arg]	*T*GG = > *C*GG	G^b^	0.1
*VEGFA*	6p 12	SNV	963C > T	*rs3025039*	UTR-3	C/T	G	na	na	T	0.149
*eNOS*	7q36	SNV	Glu298Asp (894 T > G)	*rs1799983*	Missense	G/T	G	E[Glu] = > D[Asp]	GA*T* = > GA*G*	T	0.197
*TH*	11p15.5	SNV	Va181Met (7085G > A)	*rs6356*	Missense	A/G (rev)	G	V]Val] = > M[Met]	*G*TG = > *A*TG	T^b^	0.419

^a^Minor Allele Frequency from 1000 Genomes Phase I Genotype data released in May 2011

^b^For some SNPs, testing providers detect the genotype from the opposite strand of DNA. In such cases “A” is to be replaced by “T” and “G” by “C” (and vice-versa)

*DIV* deletion/insertion variant

*SNV* single nucleotide variant

**Table 2:** Primer pair sequences and PCR conditions of the studied genetic marker

Gene variant	NCBI rsID	Primer sequence	PCR cycle	PCR cycling conditions	RE	RE Condition	Agarose (%)	Allele sizes (bp)
*EDN1* 9456G > A	*rs2071942*	F: 5’ CAAACCGATGTCCTCTGTA 3’	35	I 95 °C 5’, D 95 °C 1’, A 52 °C 1’, E 72 °C 2’, FE 72 °C 7’	*Taq* ^*α*^I	65 °C, 2 h	1.8	G: 150,208
		R: 5’ ACCAAACACATTTCCCTATT 3’						A: 358
*EDN1* 3A/4A (−134 del)	*rs1800997*	F: 5’ GCTGCCTTTTCTCCCCGTTTAA 3’	30	I 95 °C 2’, D 95 °C 30”, A 57.2 °C 30’, E 72 °C 20”, FE 72 °C 5’	*Bs1I*	5 °C O/N	3	3A:221
		R: 5’ CAAGCCACAAACAGCAGAGA 3’						4A:197, 24
*SCNN1B* Thr594Met (1781C > T)	*rs1799979*	F: 5’ ACCGTGGCCGAGCTGGTGGAG 3’	35	I 94 °C 5’, D 94 °C 30’, A 67.5 °C 1.5’, E 72 °C 30”, FE 72 °C 5’	*NlaIII*	37 °C O/N	1.5	T: 226
		R: 5’ CAGTCTTGGCTGCTCAGTGAG 3’						C: 117,109
*ADRB2* Arg16Gly (46A > G)	*rs1042713*	F: 5’-CTTCTTGCTGGCACGCAAT-3’	30	I 94 °C 10’, D 94 °C 1’, A 57 °C 1’,72 °C 1’, FE 72 °C 5’	*BsrDI*	65 °C 21/2 hr	3	A:131,56,14
		R: 5’-CCAGTGAAGTGATGAAGTAGTTGG-3						G: 14, 23, 56, 108
*ADBR2* Gln27Glu (79G > C)	*rs1042714*	F: 5’-GGCCCATGACCAGATCAGCA-3’	30	I 94 °C 10’, D 95 °C 1’, A 63 °C 1’, E 72 °C 1’, FE 72 °C 5’	*Fnu4HI*	37 °C, 3 h	3	C: 229, 97, 27
		R: 5’-GAATGAGGCTTCCAGGCGTC-3’						G: 174, 97, 55, 27
*ADBR3* Try64Arg (5387 T > C)	*rs4994*	F: 5’-CAATACCGCCAACACCAGTGG-3’	30	I 95 °C 10’, D 95 °C 30”, A 60 °C 30’, E 72 °C 30”, FE 72 °C 5’	*MspI*	37 °C, O/N	3	T: 99, 54
		R: 5’-GGTCATGGTCTGGAGTCTCG-3’						C: 70, 54, 29
*VEGF* 963C > T	*rs3025039*	F: 5’-AGGAAGAGGAGACTCTGCGCAGAG	30	I 94 °C 2’, D 94 °C 30”, A 65 °C 40’, E 72 °C 40’, FE 72 °C 5’	*NlaIII*	37 °C, 3 h	3	C: 208
		CAGGAAGAGGAGACTCTGCGCAGAGC-3’						T: 122, 86
		R: 5’-TAAATGTATGTATGTGGGTGGGTGTGTCTACAGGG-3’						
*eNOS* Glu298Asp (894 T > G)	*rs1799983*	F: 5’-CATGAGGCTCAGCCCCAGAAC-3’	30	I 94 °C 5’, D 94 °C 30”, A 63 °C 20’, E 72 °C 30’, FE 72 °C 10’	*MboI*	37 °C O/N	2.5	G: 206
		R: 5’-AGTCAATCCCTTTGGTGCTCAC-3’						T: 119, 87
*TH* Va181Met (7085G > A)	*rs6356*	F:5’-GGCAGAGCCTCATCGAGGAC-3’	40	I 95 °C 2’, D 94 °C 1’, A 63 °C 45’, 72 °C 2.5’, FE 72 °C 10’	*NlaIII*	37 °C O/N	2.5	G: 197
		R: 5’-AAACACCTTCACAGCTCGGGAC-3’						A: 131, 66

### Statistical analyses

Allele frequencies and genotype frequencies were calculated by allele counting. Genotypic and allele frequency differences between Ladakh natives and acclimatized sojourners were analyzed by Pearson chi-square (*χ*^2^) test and Fisher’s exact test respectively to find the statistical significance between the genotypes. Deviations from the Hardy-Weinberg equilibrium (HWE) were tested for all polymorphisms by comparing observed and expected genotype frequencies with an exact goodness of fit test. Probability (*p*) value of less than 0.006 was considered statistically significant after Bonferroni’s correction in genotype-based multiple testing (N of test was given as 8 as *SCNN1B Thr594Met (rs1799983:G > T)* was monomorphic). Specific roles of divergent alleles in relation to acclimatization were explored with various genetic models and significance was assessed using odds ratio and 95 % confidence interval.

## Results

The present study was conducted on two cohorts comprising young and healthy males from plains (lowlanders) and high altitude (natives) serving in the Indian Army. We studied the genetic variations of nine high altitude relevant SNPs and VNTR with a view to understanding the differences/similarity at candidate gene loci between the acclimatized and the adapted individuals. Although population stratification could be an issue to a certain extent, sea level volunteers in the present study were chosen both from North Indian (Indo Aryan) and South Indian (Dravidian) lineages as deployment at high altitude is non discriminatory, independent of ethnicity based criteria and population structure. The physiological characteristics of Ladakh natives and sea level sojourners is shown in Table 3. Body weight, heart rate and systolic and diastolic blood pressure was markedly lower in the Ladakh natives compared to the sea level sojourners. Arterial oxygen saturation (SpO_2_) was higher in the natives compared to the sojourners at high altitude (Table 3). Out of the nine high altitude relevant loci studied, genotype and allelic distribution of six loci viz., *EDN1 -3A/-4A VNTR, ADRB2 Arg16Gly (rs1042713:A > G), ADRB3 Trp64Arg (rs4994:T > C), eNOS Glu298Asp (rs1799983:T > G)*, *TH Val81Met (rs6356:G > A)* and *VEGF 963C > T (rs3025039:C > T)* were statistically similar between the Ladakh natives (adapted) and the sea level sojourners (acclimatized) (Table 4). *EDN1 -3A/-3A* was more frequent in both Ladakh natives (0.85) and sea level sojourners (0.77). None of the Ladakh natives were found to be homozygous for *-4A/-4A* while only one acclimatized sojourner was -*4A/-4A*. Frequency of wild allele *G* (*Glu)* of *eNOS Glu298Asp (rs1799983:T > G)* polymorphism was statistically similar in both high altitude natives and the sea level sojourners (0.81 and 0.85 respectively) and also higher in both the cohorts compared to minor allele T (*Asp)* frequency. Homozygous *CC* of *VEGF 963C > T (rs3025039:C > T)* was the predominant genotype in both natives and sea level sojourners; no homozygous *TT* for *rs3025039:C > T* was not found in Ladakh natives. *SCNN1B Thr594Met (rs1799973:G > T)* was monomorphic in both population with preponderance of homozygous *CC* genotype and *C* allele in all the individuals.

**Table 3:** Physiological characteristics of Ladakh high altitude natives (HAN) and sea level sojourners (Accl _LLD)

Characters	HAN	Accl_LLD	*p* value
Altitude	≥3400	Sea level	
Age (yrs)	28.71 ± 6.2	28.16 ± 6.79	0.699
Body Weight (kg)	60.79 ± 5.81	65.82 ± 5.9	2.63E-05
HR (beats/m)	73.05 ± 7.99	85.76 ± 16.49	1.7E-05
SBP, mm Hg	115.67 ± 8.51	122.32 ± 10.63	0.00084
DBP, mm Hg	77 ± 6.56	82.02 ± 9.34	0.002
S_P_O_2_ (%)	91.22 ± 2.51	88.33 ± 6.4	0.002

Results are mean ± standard deviation (S.D). *p* value is based on unpaired *t*-test

*HR* heart rate, *SBP* systolic blood pressure, *DBP* diastolic blood pressure, *SpO*_*2*_ arterial oxygen saturation

**Table 4:** Genotypic and allelic frequencies of the genetic markers in the Ladakh high altitude natives (HAN) and sea level sojourners (Accl _LLD)

SNP	Variant	Genotype	Genotype Frequency		*p*	HWE^a^ *p*		Allele	Allele Frequency		Fisher exact *p*
			HAN	Accl_LLD		HAN	Accl_LLD		HAN	Accl_LLD	
*rs2071942*	*EDN1 9465G > A* ^*b*^	*GG*	0.439	0.361	***0.005***	0.34	0.01	*G*	0.681	0.534	**0.01**
		*GA*	0.484	0.347				*A*	0.318	0.465	
		*AA*	0.075	0.291							
*rs10478694* ^c^	*EDN1 3A/4A*	-3A/-3A	0.857	0.771	0.343	0.54	0.97	*-3A*	0.928	0.878	0.06
		*-3A/-4A*	0.142	0.214				*-4A*	0.071	0.121	
		*-4A/-4A*	0	0.014							
*rs1042713*	*ADRB2 Arg16Gly* (46A > G)	*GG*	0.238	0.305	0.559	0.54	0.51	*G*	0.507	0.569	0.3
		*AG*	0.537	0.527				*A*	0.492	0.43	
		*AA*	0.223	0.166							
*rs1042714*	*ADRB2 Gln27Glu* (79G > C)^*d*^	*CC*	0.612	0.338	***0.005***	0.57	0.04	*C*	0.777	0.626	***0.002***
		*CG*	0.354	0.577				*G*	0.222	0.373	
		*GG*	0.032	0.084							
*rs4994*	*ADRB3 Try64Arg* (5387 T > C)	*TT*	0.805	0.75	0.077	0.01	0.22	*T*	0.88	0.875	0.14
		*CT*	0.149	0.25				*C*	0.119	0.125	
		*CC*	0.044	0							
*rs3025039*	*VEGF 963C > T*	*CC*	0.91	0.83	0.302	0.7	0.56	*C*	0.955	0.908	0.06
		*CT*	0.089	0.154				*T*	0.044	0.091	
		*TT*	0 (0)	0.014							
*rs1799983*	*eNOS Glu298Asp* (894 T> G)	*GG*	0.723	0.666	0.582	0.69	0.71	*G*	0.853	0.815	0.08
		*GT*	0.261	0.291				*T*	0.146	0.187	
		*TT*	0.015	0.041							
*rs6356*	*TH Val81Met* (7085G > A)	*AA*	0.461	0.45	0.726	0.9	0.36	*A*	0.676	0.659	0.76
		*GA*	0.43	0.5				*G*	0.323	0.34	
		*GG*	0.107	0.091							

^a^Hardy-Weinberg Equilibrium; ^b^amplification failed in three HAN samples; ^c^this rsID has been removed from the reference database and merged into rs1800997; ^d^amplification failed in 3 HAN and 1 sojourner sample

Genotypic and allele frequency distribution of two loci viz., *EDN1 9465G > A (rs2071942:G > A)* and *ADRB2 Gln27Glu (rs1042714:G > C)* were significantly different between Ladakh natives (adapted) and the sea level sojourners (acclimatized). Genotypic frequency of *GG:GA:AA* of *EDN1 9465G > A (rs2071942:G > A)* in Ladakh population was observed to be 0.439: 0.484:0.075 compared to 0.361:0.347:0.291 in sea level sojourners (*p* < 0.05) and allele frequency *G:A* was 0.681:0.318 in Ladakh natives compared to 0.548:0.465 in sojourners (*p* = 0.01) (Table 4). Higher frequency of wild type homozygous *GG* and heterozygous *GA* genotype of *EDN1 9465G > A (rs2071942:G > A)* and lower frequency of mutant homozygous *AA* genotype was thus observed in the Ladakh natives compared to the acclimatized sea level sojourners. For *ADRB2 Gln27Glu (rs1042714:G > C)* polymorphism*,* homozygous *CC (Gln27Gln)* genotypic frequency was higher in Ladakh natives (0.612) compared to sea level sojourners (0.338) while heterozygous *CG (Gln27Glu)* genotypic frequency was lower in the natives (0.354) compared to acclimatized sojourners (0.577). Allele *C* (*Gln27*) frequency was also higher in the Ladakh natives compared to acclimatized sojourners (p < 0.05). On our exploration of specific role of *EDN1 9465G > A (rs2071942:G > A)* in relation to acclimatization with the various genetic models, mutated *A* allele (*AA* genotype) was observed to be associated with being less favorable during acclimatization compared to *G* allele carriers (*GG* + *GA* genotypes) (*AA* vs *GA + GG*: Odds Ratio = 5.02; 95 % Confidence Interval 1.7 to 14.26, p = 0.0024, under the recessive genetic model of inheritance) while for *ADRB2 Gln27Glu (rs1042714:G > C),* carriers of mutated *G* allele (*GG* + *GC* genotypes) were found associated with being less favorable during acclimatization compared to those who had wild homozygous *CC* genotype (*GG* + *GC* vs. *CC*: Odds Ratio = 3.1; 95 % Confidence Interval 1.5 to 6.3, *p* = 0.0018, under the dominant genetic model of inheritance)

## Discussion

Ethnically diverse Indian population with distinct genetic background [44] provides a unique opportunity for studying genetic markers for high altitude acclimatization (sea level sojourners) *vis a vis* adaptation (high altitude natives). While the high altitude native recruits are adapted to the high altitude hypoxic environment, the sea level recruits are also operationally effective at high altitude post-acclimatization. Genetic information on high altitude Ladakh native population is limited. Ladakh natives are generally quite different from rest of India population; the faces and physique of the Ladakh natives and the clothes that they wear are more akin to those of Tibet and Central Asia than India. Through the present study, the genotypic and allele frequency distributions of *EDN1 9465G > A (rs2071942:G > A)*, *ADRB2 Arg16Gly (rs1042713:A > G), ADRB3 Trp64Arg (rs4994:T > C)*, *TH Val81Met (rs6356:G > A), SCNN1B Thr594Met (rs1799983:G > T)* and *VEGF 963C > T (rs3025039:C > T)* are being reported for the first time from the high altitude natives from Ladakh.

Of the nine high altitude relevant polymorphic loci studied in the sea level sojourners (acclimatized) and the high altitude natives (adapted), we noted statistically similar genotypic and allele frequencies in six loci viz., *ADRB2 Arg16Gly (rs1042713:A > G), ADRB3 Trp64Arg (rs4994:T > C), EDN1 -3A/-4A VNTR, eNOS Glu298Asp (rs1799983:T > G)*, *TH Val81Met (rs6356:G > A)* and *VEGF 963C > T (rs3025039:C > T)* in both the cohorts. This similarity may be suggestive of probable advantageous effect of the said polymorphic profiles as well as commonality in gene regulatory pathways in which these gene products function both during process of acclimatization and adaptation in high altitude environment, although the molecular signatures of the biological pathways remain to be elucidated. *ADRB2 Arg16Gly (rs1042713:A > G)* has been shown to be associated with improved oxygen delivery during altitude acclimatization [33]. Snyder and coworkers [45] showed an association between adenine (A) at position 46 (amino acid position 16) and lung fluid accumulation at high altitude while another study did not show association of this polymorphism with acute mountain sickness in Nepalese population [46]. The genotype frequency of *Arg16Gly (rs1042713:A > G)* observed in sea level acclimatized sojourners in present study is in agreement with the genotype frequency reported by Stobdan and coworkers [47] from sea level Indian individuals who were resistant to high altitude pulmonary edema (HAPE). Statistically similar as well as higher frequency distribution of wild allele *G* (*Glu*) of *eNOS Glu298Asp (rs1799983:T > G*) polymorphism in both high altitude natives of Ladakh and the sea level acclimatized sojourners observed in the present study compared to the T (*Asp*) allele suggests an advantageous role of *G* (*Glu*) allele for high altitude acclimatization similar to the adaptative advantage in the natives. Higher frequency of *Glu* allele of *eNOS Glu298Asp (rs1799983:T > G)* in the high altitude natives was reported earlier [21, 48] and suggested to be beneficial in high altitude environment through increased production of NO. Higher exhaled NO has been observed in Tibetan and Bolivian Aymara population [22, 49]. Significantly higher frequency of *Glu* allele was also reported from Quechua of the Andean *altiplano* [50] and Sherpas from the trans Himalayan region [51]. Interestingly, in Indian sojourners [48], Japanese [52] and Chinese [53] population, the *Asp* (*T*) allele and genotypes *Glu298Asp (GT)* and *Asp298Asp (TT)* were shown to be associated with high altitude pulmonary edema.

Plasma VEGF level was demonstrated to be changed during acclimatization [42]. Homozygous *CC* of *VEGF 963C > T (rs3025039:C > T)* was also reported to be associated with decreased risk of acute mountain sickness (*CC* versus *CT/TT*) [54]. Similarity in genotypic frequency of *VEGF 963C > T (rs3025039:C > T)* in acclimatized sojourners and adapted natives in the present study, with predominant genotype being homozygous *CC,* reiterates probable advantage of *CC* genotype and *C* allele in high altitude environment both during acclimatization and adaptation. In the present study we also observed statistically similar genotypic and allele frequency of *TH Val81Met (rs6356:G > A)* between the sea level acclimatized lowlanders and the high altitude natives. Sustained hypoxia, as seen during high altitude sojourn, was shown to be associated with substantial *in vivo* up regulation of *TH* gene in the caudal brain stem and increased catecholamine turnover that was further associated with adaptive ventilator mechanisms essential for respiratory homeostasis during hypoxia [39, 40]. Similarity in *TH* polymorphism in the acclimatized cohort with the adapted cohort suggests respiratory homeostasis during acclimatization to hypoxia. Contrasting reports, however, persist regarding *TH* polymorphism: while its association was reported with development of high altitude pulmonary edema through inadequate hypoxic ventilatory response [55], in a subsequent study in the Japanese population no such association was found [56].

Polymorphisms in two loci viz., *EDN1 9465G > A (rs2071942:G > A)* and *ADRB2 Gln27Glu (rs1042714:G > C)* were observed to be significantly different between the Ladakh natives and the sea level acclimatized sojourners in the present study. Frequency of homozygous *GG* genotype and *G* allele of *EDN1 9465G > A (rs2071942:G > A)* was significantly higher in the Ladakh natives compared to sea level acclimatized sojourners. High altitude natives of Ladakh have been reported to have reduced plasma levels of endothelin and *EDN1* gene expression [57] along with over expression of longer repeats of *EDN1* -*3A/-3A*, *GG* and *Lys198Lys* genotypes [24] compared to sea level sojourners. In this context, it would be pertinent to highlight that elevated levels of endothelin [58] as well as higher expression of endothelin converting enzyme (*ECE1*) was found in lowland individuals who developed high altitude pulmonary edema (HAPE) compared to acclimatized lowlanders on acute induction to high altitude [59] while no such differential gene expression was noted between the acclimatized lowlanders and the adapted natives (GEO Accession number: GSE52209). Endothelin-1 augments capillary hydrostatic pressure during high altitude pulmonary edema susceptibility [29] and venous endothelin-1 plasma level has been seen to be higher in HAPE susceptible compared to mountaineers who are resistant to high altitude hypoxia [28]. As an extension of our understanding of role of *rs2071942* in relation to acclimatization with the various genetic models, we noted mutated *A* allele (*AA* genotype) being less favorable during acclimatization compared to *G* allele carriers under recessive model of inheritance. Thus *GG* genotype and *G* allele of *rs2071942* is beneficial for acclimatization*.* It may be also pertinent to mention here that significant difference in genotypic frequency of *EDN1 9465G > A (rs2071942:G > A)* between acclimatized sojourners and individuals who developed HAPE, with *GG* genotypic frequency being significantly lower in HAPE individuals has been observed by us (data not shown). It is probable that higher *GG* genotypic frequency of *EDN1 9465G > A (rs2071942:G > A)* in Ladakh natives compared to acclimatized sojourners as observed in the present study, coupled with over expression of longer repeats of *EDN1* -*3A/-3A*, *GG* and *Lys198Lys* genotypes [24] confers further selective advantage for high altitude dwelling. The difference in *rs2071942* frequency observed in the two cohorts would be of functional significance as serum endothelin level is affected by the *EDN1* genetic variability; *AA* genotype of *rs2071942* has been observed to be associated with higher endothelin level and predisposition to coronary artery disease [60]. *rs2071942,* which is an intronic variant at intron 4 (*G/A*, chromosome position 12294760, GRCh 38.2, NCBI *Homo sapien* Annotation release 107) has been reported to be in tight linkage disequilibrium (LD) with a functional exonic SNP, *rs5370* (exon 5, *Lys198Asn*, chromosome position 12296022, GRCh 38.2, NCBI *Homo sapien* Annotation release 107) with rare *A*-*Asn* haplotypes being associated with higher hazard ratio in cardiomyopathy compared to the ‘protective’ effect of the common *G-Lys* haplotypes [61]. Strong LD was also reported to be present between *rs5370, rs2071942* and *rs1800543* variant in intron 3 along with three additional intronic SNPs *rs2070699*, *rs5369* and *rs6413479* which define the same haplotype block [62]*.* It would, however, be imperative to perform transfection studies with various haplotype-expressing constructs, electrophoretic mobility shift assays and binding studies to ascertain whether *rs2071942* is functionally different between the adapted and the acclimatized cohorts.

Moore and coworkers [63] identified *EDN1* as one of the hypoxia inducible factor (HIF) pathway genes that was candidate target of positive selection involved in regionally restricted adaptation to high altitude. Systematic scanning of the *EDN1* locus in high altitude natives, sojourners and the maladapted individuals should unravel the high altitude hypoxic signature in this genomic region and thereby its role in acclimatization and adaptation. The emerging aspects of epigenetic regulation of *EDN1* by histone modification [64], microRNA (miRNA)-mediated regulation [65], methylation of CpG islands and gene silencing [66] and significance of these mechanisms in context of genetic polymorphism in the overall physiological control of *EDN1* in adaptation and acclimatization will require further investigation.

Apart from *EDN1 9465G > A (rs2071942:G > A),* we also noted higher frequency of wild type homozygous *CC* genotype and *C* allele of *ADRB2 Gln27Glu (rs1042714:G > C)* in Ladakh natives compared to the sea level acclimatized sojourners (*p* < 0.05). This indicates that *C* allele and *CC* genotype confers acclimatization as well as adaptation benefit over the *CG + GG* genotype in the high altitude environment. *rs1042714* (*79C > G, Gln27Glu*) is a coding polymorphism causing non synonymous changes in the amino acid. Being a functional SNP, difference in genotype and allele frequency of *rs1042714* observed in the present study in the cohorts would be of functional significance. *ADRB2* function was shown to be altered by the *rs1042713* and *rs1042714* allele mutations by altering the amino acid sequence in the extracellular N-terminus of the *ADRB2* [67]. On exploring specific role of *rs1042714* in relation to acclimatization with the various genetic models, we noted that carriers of *G* allele (*GG* + *GC* genotypes) were less favored for acclimatization compared to those who had wild homozygous *CC* genotype (Odds Ratio = 3.1; 95 % Confidence Interval 1.5 to 6.3, p = 0.0018). In the Indian population, haplotypes of *ADRB2* consisting of SNPs *Arg16Gly(46A > G)* and *Gln27Glu (79C > G)* showed a greater power for predicting high altitude pulmonary edema with alleles *A46* and *G79* being associated with increased receptor sensitivity [47]. Involvement of natural selection in Ladakh native highlanders is indicated by divergence in *rs1042714* allele frequency between the two cohorts in the present study. In this context it is stated that the Ladakh natives are of East Asian (Tibetan) origin while lowland Indians have diverged from East Asian population and more connected to western Eurasian population. 1000 Genome Phase 3 data has shown minor allele (*G*) frequency of *rs1042714* to be 0.073 in East Asian, 0.410 in European and 0.19 in South Asian population [68]. Frequency of *rs1042714* observed in the Ladakh natives (0.222) and in the acclimatized sojourners (0.373) is included in the range reported from the 1000 Genome project. In high altitude Quechua population, the *C* allele was shown to be monomorphic [69]. Role of beta adrenergic receptors in the high altitude environment is well documented. These receptors are present in the airway smooth muscle cells with beta-2-subtypes as the dominant receptors (70 %) in the lungs [31]. Role of ADRB2 in regulating lung fluid clearance is well defined [32]. In the present study we noted *CC* genotype of *rs1042714* to be beneficial at high altitude, however, in Kyrgyz highlanders, higher frequency of homozygous *CC* genotype of *ADRB2 Gln27Glu (rs1042714:G > C)* was reported to be associated with high altitude pulmonary hypertension [70]. In the Indian context, on the contrary, it was suggested that hypobaric hypoxia may have a protective effect from developing hypertension which was based on a study from Indian highlanders from Spiti valley (a high altitude region in Himachal Pradesh, terrestrial elevation 3000 m to 4200 m) [71]. Nevertheless, as ‘lifestyle diseases’ including hypertension are on the rise in Ladakh natives (www.reachladakh.com/archive_details.php?pID=2610 ), it may be worthwhile to survey the prevalence of high altitude pulmonary hypertension in Indian highlanders and correlate with *ADRB2 Gln27Glu (rs1042714:G > C)* frequency in a large cohort. Moreover as *ADRB2 Gly16Arg* (*46 A > G*, *rs1042713*) and *Gln27Glu* (*79 C > G*, *rs1042714*) are located near the receptor ligand binding site [72], it would be worthwhile to assay functional importance of these polymorphisms through ligand induced conformational changes for understanding their role, if any, in acclimatized and adapted cohorts.

In view of the observation that *rs2071942* and *rs1042714* were significantly different between the adapted and the acclimatized cohort, we further explored how these variants affected genes, transcripts and protein sequences using Ensembl Variant Effect Predictor (VEP) (Ensembl release 81-July 2015) [73] which uses tools such as SIFT and PolyPhen and includes the Ensembl regulatory features, miRNA targets and other relevant regulatory regions. It was interesting to note that the VEP found overlap of *rs2071942* and *rs1042714* with regulatory features (core base pairs: chromosome 6_12286106-12297305 and chromosome 5_148825705-148830504 respectively). The output of the Variant Effect Predictor is shown in Table 5. The regulatory feature type for *rs2071942* was promoter flanking region with highest score for purine rich 5’-GAGGAA-3’ PU.1 (Winged Helix-Turn-Helix) element (PWM ID MA0080.3, Score 11.56) and CTCF enriched element (PWM ID MA0139.1, Score 9.195). For *rs1042714,* a CTCF-binding motif in the promoter region was predicted. Presence of these motif sequences were confirmed by alignment on the *Homo sapien* chromosome 6: *EDN1* and chromosome 5: *ADRB2* GRCh38.p2 Primary Assembly. Table 6 lists the motif information for both the variants. Presence of these motifs in the promoter flanking and promoter region of *EDN1* and *ADRB2* respectively suggest, but not yet proved, further transcriptional regulation of these genes. Endothelin 1 transcription is highly regulated and there are multiple regulatory elements on the *EDN1* promoter which govern the *EDN1* gene activity (reviewed in [74]). It would be important to ascertain the functions of PU.1 and CTCF-binding regulatory motifs in high altitude adaptation and acclimatization. In earlier studies, *rs2071942* was also predicted not to localize to any known functional splicing or regulatory site or alter splicing thereby lacking in any obvious functional effect [61]. Softberry FSPLICE [75] revealed a AG splice site (tctacAGgtaga) at position 12294650–12294662 and a GT splice site (tacagGTagatt) at position 12294652–12294664, 98–110 bp upstream of *rs2071942* in our analysis*.* These *in silico* predictions of splice variants are required to be supported by laboratory evidences for finding effects, if any, in high altitude environment.

**Table 5:** Ensemble Variant Effect Predictor (VEP) result

Uploaded variation	Location	Allele	Consequence	Impact	Symbol	Gene	Feature type	Feature	Biotype	Exon	Intron	cDNA position	CDS position	Protein position	Amino acids	Codons	SIFT	PolyPhen	GMAF	Clinical significance
rs2071942	6:12294760-12294760	A	Intron_variant	MODIFIER	*EDN1*	ENSG00000 78401	Transcript	ENST00000 379375	Protein_coding	-	4	-	-	-	-	-	-	-	A: 0.2558	-
rs2071942	6:12294760-12294760	A	Regulatory_region_variant	MODIFIER	-	-	Regulatory feature	ENSR00001 211220	Promoter_flanking_region	-	-	-	-	-	-	-	-	-	A: 0.2558	
rs1042714	5:148826910-148826910	C	Missense_variant	MODERATE	*ADRB2*	ENSG00000169252	Transcript	ENST00000305988	Protein_coding	1	-	1666	79	27	E/Q	GAA/CCA	tolerated (0.47)	benign (0.008)	G:0.2043	risk_factor
rs1042714	5:148826910-148826910	C	Regulatory_region_variant	MODIFIER	-	-	Regulatory feature	ENSR00001293358	Promoter	-	-	-	-	-	-	-	-	-	G:0.2043	risk_factor

**Table 6:** Regulatory region consequences predicted by Ensemble Variant Effect Predictor

rsID	Feature	Motif	PWM ID	Score
*rs2071942*	ENSR00001211220	CTCF	MA0139.1	9.195
		cjun	MA0303.1	7.74
		CTCF	MA0139.1	7.245
		PU.I	MA0080.3	11.569
		PU.I	PB0058.1	9.826
*rs1042714*	ENSR00001293358	CTCF		

*PWM* position weight matrix

Considering that the genotype frequencies of six out of nine high altitude relevant loci were not significantly different between the acclimatized lowlanders and the high altitude natives in the present study, what would the chances be of finding some genes not anticipated to play a role in high altitude adaptation that may differ in this type of cohort? For seeking this information, we looked into the Indian Genome Variation Consortium dataset (2008) [76] for genotype frequencies of a few genes (not anticipated to play a role in high altitude adaptation). The Indian Genome Variation Consortium dataset (2008) consists of frequency profiles from diverse endogamous Indian population of 405 functional candidate SNPs from 75 disease and drug response related genes and a 5.2 Mb chromosome 22 genomic region. The study demonstrated large level of genetic divergence between groups of Indian population that clustered largely on basis of ethnicity and language [76]. Analysis of genetic variation through System Structure revealed five major groups in the Indian population with the Indo European speaking large and special population and the Tibeto-Burman speaking special population of the Himalayan belt as two near homogenous groups [76]. Quantitative measures of differentiation (F_ST_) between pairs of Tibeto-Burman and Indo-European population and between Tibeto-Burman and Dravidian was high indicating population differentiation while F_ST_ value between Indo-European and Dravidian population varied between 0.000 to 0.044 [76]. From this dataset, we randomly chose a few SNPs which were mostly associated with disease and drug response (SNPs not so far reported to be associated with high altitude adaptation) from lowlander population groups comprising Indo-European large population (IE-W-LP3, inhabitants of Rajasthan) and the Dravidian large population (DR-S-LP2, inhabitants of Andhra Pradesh) comparable to the lowlander group in the present study and the highlander population of the Himalayan belt represented by the Tibeto-Burman speaking special population (TB-N-SP2, inhabitants of Jammu and Kashmir) comparable to the high altitude natives in the present study. Genotype frequencies of the SNPs in the dataset were recalculated and compared through χ^2^test. Significant statistical difference in the chosen SNPs was observed between the Tibeto-Burman speaking population of the Himalayan belt and the lowlanders (Additional file 1: Table S1). Based on this analysis, it is therefore reasonable to believe that the six out of nine high altitude relevant loci in acclimatized sojourners that show statistical similarity with high altitude natives viz., *ADRB2 Arg16Gly (rs1042713:A > G), ADRB3 Trp64Arg (rs4994:T > C), EDN1 -3A/-4A VNTR, eNOS Glu298Asp (rs1799983:T > G)*, *TH Val81Met (rs6356:G > A)* and *VEGF 963C > T (rs3025039:C > T)* together with the presence of ‘beneficial’ variants of *EDN1 9465G > A (rs2071942:G > A)* and *ADRB2 Gln27Glu (rs1042714:G > C)* confer acclimatization advantage in lowlanders at high altitude.

In recent years, a growing body of work has focused on the genetic basis of high-altitude adaptation. Candidate gene approach as well as genome scans have identified several genetic variants and markers pertaining to human adaptation in high altitude Andean, Tibetan and Ethiopian population that play a role in physiological adaptation to high altitude [63, 77–82]. A limited number of candidate genes tested in high altitude native population have revealed genetic variants that underlie intra- and inter- population differences in altitude performance. In one of the first genome-wide scans for selection in high-altitude Andean population, four HIF pathway genes that are candidate targets of positive selection were identified which were highly differentiated between the high- and the low-altitude populations and could be involved in regionally restricted adaptation to high altitude viz., *EDN1* (endothelin 1), *NOS2A* (nitric oxide synthase 2), *ADRA1b* (alpha-1B-adrenergic receptor) and *PHD3* (HIF-prolyl hydroxylase 3) [63]. Bigham and coworkers [77] further identified *VEGF* (vascular endothelial growth factor), *TNC* (tenascin C), *CDH1* (cadherin 1), *EDNRA* (endothelin receptor A), *PRKAA1* (protein kinase, AMP-activated, alpha 1 catalytic subunit), *ELF2* (E74-like factor 2), *PIK3CA* (phosphoinositide-3-kinase, catalytic, alpha polypeptide) and *EGLN1* as candidate genes. In Tibetan highlanders, *EPAS1* (HIF2α), which is associated with low hemoglobin concentration, was identified [8]. A human genome scan using 998 polymorphic DNA markers found association between the markers and hypoxic adaptation in Sherpa porters living in the Solu-Khumbuarea [83].

The present study has limitation that needs to be highlighted: i) it does not provide much information whether the similarities/differences reflect chance play or suggest true differences between the two populations, ii) the underlying basal neutral variations in Ladakh population is yet to be resolved and the extent of linkage disequilibrium at or near to the studied loci remains to be ascertained, iii) from the frequency distribution of the studied polymorphisms, it is also difficult to discern if there had been any genomic admixture between the high altitude resident population of Ladakh and the lowland Indian population. The Indian Genome Variation Consortium data reported that the isolated population of the Himalayan belt (irrespective of their linguistic background) was closest to the Chinese (CHB) and Japan (JPT) population and separated from the rest of India population when compared to the HapMap dataset [76]. It has also been suggested that both Himalayans and Andeans are likely to have descended from the same ancestral population that migrated from Asia across ancient Beringea to the New World [84]; comparative genetic studies should be undertaken to get further clue. Another issue that requires to be addressed is population stratification although Indian Army deployment in high altitude is not based on ethnicity criteria or population structure. We would like to mention here that in studies conducted on ethnic recruits from Indian Army viz., the North Indian (Indo Aryan) and South Indian (Dravidian) population for addressing issues of population stratification, we did not observe statistical difference in genotype and allele frequency distribution in *eNOS Glu298Asp* (unpublished), *AGT T704C* (unpublished), *ACE* insertion *(I)/*deletion *(D)* [12] and *ACTN3 R577X* [12] between the North Indian and South Indian lowlanders. Genotypic and allele frequency similarities in other genetic markers have also been noted between the lowlanders in other studies [85, 86]. In a recent study, high level of similarity of common gene variants was reported to exit between the North Indian and South Indian population [87].

## Conclusion

In conclusion, it is stated that of the nine high altitude relevant loci studied, we found similarity in genotypic and allele frequency distribution in six of the loci between the acclimatized sea level sojourners and the adapted high altitude natives. This is suggestive of probable advantageous effect of the observed frequencies and consequent commonality in gene regulatory pathways both during acclimatization and adaptation in high altitude environment. Natural selection, furthermore, would have favored certain genes for adaptive advantage for high altitude dwelling in the natives. Identifying genetic markers beneficial for high altitude sojourn and separating the adaptive changes from the acclimatization processes is worth investigating. Exome sequencing and high resolution genome wide association studies, which are considered to be unbiased by prior assumptions regarding sequence alteration responsible for phenotypic variation, will identify more candidate genetic markers for high altitude acclimatization and adaptation. Acclimatization to high altitude has become an important preparatory process for all athletes participating in sports activity conducted above 1500 meters wherein the prevailing conditions at this elevation make physical activity difficult and performance limited [88]. A genetic predisposition score, composed of six polymorphisms viz., *rs833070* (*VEGFA*), *rs4253778*(*PPARA*), *rs6735530* (*EPAS1*), *rs4341* (*ACE*), *rs1042713* (*ADRB2*) and *rs1042714* (*ADRB2)* was significantly associated with a diminished maximal aerobic capacity in response to acute hypoxia in lowlanders [89]. Ability to identify common/different pathways between the high altitude acclimatized and the high altitude adapted holds the key to identification of genetic components, discovery of acclimatization/adaptation markers, understanding mechanisms of acclimatization/adaptation as well as pharmacologic prophylaxis, all of which will be important for maintenance of health, performance, capability and morale of individuals who ascend to high altitudes for many reasons.

## Additional file

Additional file 1: Table S1. Statistical analysis of SNPs not yet reported to play a role in high altitude environment. (DOCX 15 kb)
