# Supplementary material for: Polymorphism profiling of nine high altitude relevant candidate gene loci in acclimatized sojourners and adapted natives
Source: BMC Genet. 2015 Sep 15;16:112. doi: 10.1186/s12863-015-0268-y (PMC4572652; doi:10.1186/s12863-015-0268-y)
Supplement: Additional file 1: Table S1. — Statistical analysis of SNPs not yet reported to play a role in high altitude environment. (DOCX 15 kb) [file 12863_2015_268_MOESM1_ESM.docx]

**Additional Table 1:** Statistical analysis of SNPs not yet to reported to play a role high altitude environment.

_________________________________________________________________________________________________________________________________________________

Gene Gene Name SNP Syn/ Association^†^ Genotype^†^ Genotype Frequency^†^ p -value

type Non Syn TB_N_SP2 IE_W_LP3 DR_S_LP2 TB vs IE TB vs DR IE vs DR

(n=46) (n=46) (n=46)

_________________________________________________________________________________________________________________________________________________

ABC11 ATP-binding cassette Exon Syn Drug response gene, Cancer TC 0.393 0.526 0.727 0.001 0.002 0.029

*(rs246221)* transporter sub-family V/V and Aging, Metabolic Disorders TT 0.250 0.000 0.045

C member 11 CC 0.357 0.474 0.227

PARK2 Parkin RBR E3 ubiquitin Exon Non Syn Neuropsychiatric AG 0.167 0.000 0.000 0.000 0.000 0.398

*(rs1801474)* protein ligase S/N AA 0.233 0.950 0.919

GG 0.600 0.050 0.081

XRCC5 X-Ray Repair Complementing Intron NA Cancer and Aging CT 0.000 0.071 0.065 0.014 0.023 0.976

*(rs3821104)* Defective Repair In Chinese CC 0.824 0.571 0.587

Hamster Cells 5 TT 0.176 0.357 0.348

P53 Phosphoprotein p53 Intron NA Cancer and Ageing GA 0.824 0.727 0.657 0.214 0.077 0.20

*(rs1625895)* GG 0.000 0.000 0.057

AA 0.176 0.273 0.286

1L4R Interleukin 4 Receptor Exon NA Cancer and Ageing AG 0.371 0.227 0.154 0.016 0.012 0.553

*(rs38832)* AA 0.057 0.273 0.231

GG 0.571 0.500 0.615

ATP7B ATPase, Cu++ Transporting, Exon NA Infection and Immune GA 0.161 0.462 0.159 0.000 0.284 0.003

*(rs732774)* Beta Polypeptide Malfunction GG 0.516 0.154 0.364

AA 0.323 0.385 0.477

MTHFR Methylene Tetra Hydro Exon Non Syn Cancer and Aging, Neuropsychiatric AG 0.000 0.227 0.154 0.000 0.000 0.553

*(rs2274976)* Folate Reductase R/Q Cardiovascular, Infection and AA 0.914 0.273 0.231

Immune, Metabolic Disorders GG 0.066 0.500 0.615

CASP8AP2 Caspase 8 Associated Protein 2 Intron NA Cancer TC 0.333 0.047 0.053 0.000 0.000 0.976

*(rs457665)* TT 0.131 0.581 0.605

CC 0.533 0.370 0.342

COPA Coatomer Protein Complex, Promoter NA Other TG 0.091 0.302 0.565 0.016 0.000 0.027

*(rs1886417)* Subunit Alpha TT 0.273 0.140 0.130

GG 0.636 0.558 0.304

GDAP1 Ganglioside Induced Intron NA Other AC 0.036 0.211 0.513 0.000 0.000 0.000

*(rs12545409)* Differentiation AA 0.750 0.289 0.359

Associated Protein 1 CC 0.214 0.500 0.128

S100B S100 Calcium Binding Exon NA Cancer TC 0.029 0.045 0.051 0.012 0.237 0.402

*(rs9722)* Protein B TT 0.412 0.705 0.564

CC 0.559 0.250 0.385

Pde5A Phosphodiesterase 5A, Intron NA Others AG 0.147 0.000 0.044 0.000 0.002 0.299

*(rs1010739)* CGMP-Specific AA 0.324 0.791 0.689

GG 0.529 0.205 0.267

________________________________________________________________________________________________________________________________________________

†Association and genotypic frequency taken from Indian Genome Variation Consortium, 2008 [76]
